# Temporally Resolved Single-Cell RNA Sequencing Reveals Pathogenesis and Immune Responses in Intracerebral Bacille Calmette–Guérin (BCG) Infection

**DOI:** 10.3390/pathogens15050531

**Published:** 2026-05-14

**Authors:** Shiqi Xie, Huiling Wang, Shaoqiong Huang, Yawen He, Ying Zhang, Shuqi Yang, Xuejiao Huang, Yang Ren, Xiao-Yong Fan, Zhidong Hu, Feng Li

**Affiliations:** 1 Shanghai Public Health Clinical Center, Fudan University, 2901 Cao Lang Road, Jinshan District, Shanghai 201508, China; 2 Department of Ultrasound, Shanghai General Hospital, Shanghai Jiao Tong University School of Medicine, No. 100 Haining Road, Hongkou District, Shanghai 200080, China; 3 Shanghai Institute of Infectious Disease and Biosecurity, Fudan University, 130 Dong An Road, Xuhui District, Shanghai 200032, China

**Keywords:** BCG vaccine-inject mouse model, intracerebral mycobacterial infection, single-cell RNA sequencing, ependymal cell, immune response

## Abstract

**Background:** In some children with immunodeficiency, Bacille Calmette–Guérin (BCG) vaccination can lead to dissemination and severe infection, including severe intracranial infection, called disseminated BCG disease (BCGosis), which is characterized by high rates of disability and mortality. However, the specific routes by which BCG crosses CNS barriers and the patterns of temporal remodeling of the CNS immune microenvironment during infection have yet to be fully elucidated. **Methods:** Mice were infected with BCG through tail vein injection to construct an intracerebral mycobacterial infection mouse model, wherein the brain was collected and analyzed using single-cell RNA sequencing. We profiled temporal transcriptomic changes in cell populations, pathways, and cell–cell communication associated with anti-mycobacterial activity and inflammation-induced disturbance of physiological brain activities. **Results:** After BCG was injected via tail vein, histopathological images and cultured colonies of brain tissue confirmed successful brain infection. Then, whole-brain tissue was dissected for 10× Genomics single-cell sequencing, and we acquired 15 cell types. Dysfunction and inflammatory responses were observed in endothelial and ependymal cells. Infection induced dynamic state transitions in microglia, enabling their differentiation into disease-related and interferon-responsive states. Along with peripheral immune cells, microglia formed temporally structured communication networks that mediated early events such as chemokine recruitment and inflammatory storms, and facilitated late-stage immune checkpoint upregulation. **Conclusions:** This study proposes BCSFB as a possible pathway of mycobacteria invasion and reveals the temporality of immune response processes in the pathogenesis of intracerebral mycobacterial infection.

## 1. Introduction

Bacille Calmette–Guérin (BCG) is a live attenuated strain of Mycobacterium bovis and the only clinically licensed vaccine against tuberculosis (TB). As a cornerstone of global TB control, BCG vaccination has substantially reduced the burden of childhood tuberculous meningitis (TBM) and miliary tuberculosis [1,2]. However, under certain circumstances, BCG vaccination may facilitate BCG-associated diseases, with a significantly higher risk in HIV-infected children [3]. In rare cases, BCG can invade the bloodstream, leading to systemic dissemination and involvement of multiple organs, including the skin, lungs, liver, and even the brain [4]. This life-threatening condition is defined as disseminated BCG disease (BCGosis), characterized by a poor clinical prognosis and high mortality (up to 60%) [5]. While it predominantly affects children with primary immunodeficiencies, chronic granulomatous disease, or HIV/AIDS [6,7], it can also occur in humans without obvious immunodeficiency and in bladder cancer patients treated with BCG [8,9].

In rare cases, central nervous system (CNS) involvement is documented. BCG can spread and disrupt the blood–brain barrier (BBB) by infecting endothelial cells or through the diapedesis of infected cells. Subsequently, newly formed granulomas might rupture via necrosis, leading to meningeal and intracerebral dissemination [10]. Clinically, patients often present with fever, headache, irritability, crying, cognitive impairment, or even fall into a coma [8,11].

Advances in multi-omics have deepened our understanding of infection. However, the intricate cellular interactions within the CNS still limit our current knowledge of immunopathological mechanisms. By employing single-cell sequencing, researchers can generate single-cell atlases of infectious diseases, encompassing cell subtypes, gene expression, and intercellular interactions. Through integrating in vivo and in vitro experiments, these atlases help uncover previously unrecognized pathogenic mechanisms, while also pointing toward innovative diagnostic tools or therapeutic interventions [12].

Existing studies have explored the underlying immunopathological mechanism by establishing experimental models based on intracranial BCG infection, combined with single-cell sequencing. They revealed that CNS mycobacterial infection can trigger widespread neuroimmune activation and partial metabolic reprogramming, and ependymal cells show prominent transcriptional changes, which may be involved in hydrocephalus and neurodegeneration [13,14]. Hematogenous spread is recognized as a predominant physiological route through which pathogenic mycobacteria invade the CNS. Nevertheless, the dynamic immune landscape and molecular mechanisms that drive brain inflammatory responses during natural hematogenous BCG dissemination remain poorly characterized. In particular, single-cell-level evidence illustrating the early onset, progressive evolution, and temporal dynamics of brain immune activation after systemic BCG exposure is still lacking, including the sequential remodeling of cellular composition, functional polarization, and intercellular communication throughout the course of infection. In this study, we employed time-series single-cell RNA sequencing to dissect the dynamic alterations in the brain immune microenvironment following hematogenous BCG infection, aiming to identify key cellular subsets, molecular events, and intercellular interaction networks.

## 2. Materials and Methods

### 2.1. Bacterial Strains and Growth Conditions

All infections were performed with green fluorescent protein (GFP)-expressing BCG (GFP-BCG); the background is the BCG-Pasteur strain. GFP-BCG were grown at 37 °C in liquid Middlebrook 7H9 broth (BD Difco, Franklin Lakes, NJ, USA) supplemented with 10% (*v*/*v*) oleic acid-albumin-dextrose-catalase (OADC), 0.5% glycerol, 0.05% Tween 80 and 25 μg/mL kanamycin (Sigma, St. Louis, MO, USA), or on a solid agar plate of Middlebrook 7H11 (BD Difco) supplemented with 10% OADC and 0.5% glycerol. The preparation and quantification of single-colony suspensions were performed as outlined in a previous study [15]. BCG in the logarithmic growth phase was collected, centrifuged at 12,000× *g* for 30 s, and washed twice with PBS containing 0.05% Tween-80 (PBST). Then, the pellet was gently resuspended in PBST and centrifuged at 500× *g* for 2 min. Lastly, the supernatant was harvested as a single bacterial suspension and used for experiments after standardization of concentration based on OD600.

### 2.2. Animals and Intracerebral Mycobacterial Infection Mouse Model

The animal study was reviewed and approved by the Laboratory Animal Welfare and Ethics Committee of Shanghai Public Health Clinical Center (No. 2021-A047-01). Female BALB/c mice, 6 to 8 weeks old, were infected by the intravenous route with 2 × 10^6^ colony-forming units (CFUs) of BCG-GFP strain. Control BALB/c mice were injected with equivalent volumes of Phosphate-Buffered Saline (PBS; absin, Shanghai, China) (200 μL). At the specified time points post infection, mice were anesthetized intraperitoneal (i.p.) with 2.5% avertin (Sigma) and transcardially perfused with at least 25 mL ice-cold and sterile PBS. After dissection, the brains and lung lobes were harvested [16].

### 2.3. Measurements of Bacteria Load in Tissues

Bacterial loads in mouse brains and lungs were measured on different days post-infection (dpi). Tissues were homogenized in PBS under sterile conditions. Briefly, 100 μL of the homogenate was plated on Middlebrook 7H11 plates for the quantification of colonies. Ten-fold serial dilutions of the homogenates were prepared and added onto plates. After 21 days, CFUs were counted.

### 2.4. Intracerebral Cytokine Levels

To evaluate the inflammatory response to BCG infection, brain and lung homogenates were centrifuged to remove tissue debris, and the supernatants were sterilized through filtration. Cytokine concentrations were quantified using commercially available anti-mouse ELISA kits (Invitrogen, Carlsbad, CA, USA), following the manufacturer’s instructions. IL1β, TNF-α and IL-6 concentrations were measured by reading absorbance at 450 nm using Biotek cytation5 (Biotek, Winooski, VT, USA).

### 2.5. HE Staining

The brains and lung lobes of mice were quickly fixed in 4% paraformaldehyde solution and embedded in paraffin. They were then sectioned at a thickness of 5 μm, stained with H&E, and photographed using a TissueFAXS200 (TissueGnostics, Vienna, Austria).

### 2.6. Single-Cell Isolation of the Brain

Brain single-cell isolation was performed using a protocol previously described [17,18,19]. Mice were decapitated, and brains were placed on ice-cold Roswell Park Memorial Institute (RPMI) 1640 medium (Hyclone, South Logan, UT, USA). Brains were cut up with surgical scissors, followed by the addition of enzyme mix (10 U/mL DNAse I (Thermo Fisher, Waltham, MA, USA), 1 mg/mL collagenase (Invitrogen)) diluted in RPMI 1640 medium. Following 30 min at 37 °C, the digested tissues were then filtered through a 70 μm cell strainer (Thermo Fisher) by gently pressing with a syringe plunger. After centrifuging at 500× *g* at 4 °C for 25 min without acceleration/braking, a discontinuous 30%/70% Percoll (GE Healthcare, Little Chalfont, Buckinghamshire, UK) gradient was used to remove cell debris, myelin, and neurons, and then microglia and infiltrating leukocytes were collected from the interface. For FACS, after having been washed with PBS, the pellet was resuspended in PBS supplemented with 2% fetal bovine serum (FBS; Biological Industries, Kibbutz Beit Haemek, Israel). Total viable cell number was counted, and only samples with cell viability above 80% were used for subsequent procedures.

### 2.7. Flow Cytometry

Brain single-cell suspension was stained with Fixable Viability Stain 510 (BD Horizon, San Jose, CA, USA) at a concentration of 1:1000 for 15 min (room temperature). Subsequently, the cells were washed with PBS/2% FBS and incubated with anti-CD16/32 (clone 2.4G2, BD Pharmingen, San Diego, CA, USA) at a concentration of 1:100 in PBS/2% BSA at 4 °C for 20 min. For brain immune cell staining, the following antibodies were used [20]: anti-CD45-allophycocyanin (APC) Cyanine 7 (clone 30-F11, BD Pharmingen), anti-CD11b-fluorescein isothiocyanate (FITC) (clone M1/70, Biolegend, San Diego, CA, USA)/anti-CD11b-RY610 (clone M1/70, BD Optibuild, San Jose, CA, USA), anti-CD11c-BV605 (clone HL3, BD Horizon), anti-CD3-RB705 (clone 17A2, BD Optibuild), anti-CD4-BUV395 (clone H129.19, BD Optibuild), anti-CD19-APC (clone 1D3, TONBO biosciences, San Diego, CA, USA), anti-Ly6G-BV786 (clone 1A8, Biolegend), anti-Ly6C-PE-Cyanine7 (clone HK1.4, Invitrogen), and anti-MHC-II-BV421 (clone M5/114.15.2, BD Horizon) (all 1:100); these were added to the sample and incubated at 4 °C for 30 min. For staining for microglia, antibodies for the panel—anti-CD45-APC Cyanine 7 (clone 30-F11, BD Pharmingen), anti-CD11b-FITC (clone M1/70, Biolegend), anti-CD16/32-PerCP-Cy5.5 (clone 2.4G2, BD Pharmingen), anti-CD86-APC (clone 172.2, Invitrogen), anti-CD206-PE (clone Y17-505, BD Pharmingen), and anti-MHC-II-BV421 (clone M5/114.15.2, BD Horizon) (all 1:100)—were added, and the sample was incubated at 4 °C for 30 min. After nuclear disruption for 1 h, anti-Ki67-PE-Cy7 (clone B56, BD Pharmingen) was added, and the sample was incubated at 4 °C for 1 h. All cells were subsequently washed with PBS/2% FBS. Cells were acquired on a LSRFortessa (BD Biosciences, San Diego, CA, USA) or CytoFlex S (Beckman Coulter, Brea, CA, USA), and analyzed using FlowJo software (version 10.8.1). All percentages are of single-viable frequency, unless otherwise indicated.

### 2.8. Single-Cell RNA Sequencing Preparation

The single-cell suspensions were converted into barcode-labeled scRNAseq libraries following the manufacturer’s instructions for the Chromium Single Cell 3′ Library, Gel bead & index kit, and Chip G Kit (v3.1, 10× Genomics), aiming to recover 10,000 cells per library. Sequencing was performed with Illumina NovaSeq according to the manufacturer’s instructions.

### 2.9. Generation and Analysis of Single-Cell Transcriptomes

Raw reads were demultiplexed and mapped to the mouse reference genome by 10× Genomics Cell Ranger pipeline with default parameters. All downstream single-cell analyses were performed using Cell Ranger (7.0) and Seurat (4.4.0) [21,22], unless specifically mentioned otherwise. In brief, for each gene and each cell barcode (filtered by Cell Ranger), unique molecule identifiers were counted to construct digital expression matrices. Secondary quality control and filtering were subsequently performed using Seurat. Genes expressed in at least 3 cells and cells with at least 200 detected genes were retained. Cells with mitochondrial gene percentage > 10% and those with aberrant UMI counts were removed to eliminate dead cells and empty droplets.

After quality control, the Seurat package was used to normalize data and to perform dimensionality reduction, clustering, and differential expression. Principal component analysis (PCA) was then performed, and batch effects were removed using the Seurat integration workflow based on canonical correlation analysis (CCA) [23] for an integrated analysis of datasets. After integration, cell clustering was conducted using FindNeighbors and FindClusters with a resolution of 0.6. Two-dimensional visualization was achieved using uniform manifold approximation and projection (UMAP).

### 2.10. Cell Annotation and Subclustering Analysis

Cell types were manually annotated by identifying cluster-specific differentially expressed genes using the FindMarkers function in Seurat, followed by assignment using cell-type marker genes from published literature. Key marker genes included: microglia (*Tmem119*, *P2ry12*, and *Cx3cr1*), macrophages (*Cd163*, *Mrc1*, and *Lyz2*), astrocytes (*Gfap*, *Aldh1l1*, and *Aqp4*), oligodendrocytes (*Mbp*, *Plp1*, and *Mog*), neurons (*Rbfox3*, *Snap25*, and *Syp*), T cells (*Cd3e*, *Cd4*, and *Cd8a*), B cells (*Cd19*, *Ms4a1*, and *Cd79a*), neutrophils (*S100a8*, *S100a9*, and *Csf3r*), NK cells (*Nkg7*, *Klrb1c*, and *Gzma*), endothelial cells (*Cldn5*, *Flt1*, and *Pecam1*), and choroid plexus epithelial cells (*Ttr*, *Enpp2*, and *Folr1*). The expression distribution of key marker genes across clusters was visualized using violin plots and feature plots to confirm cell-type identities.

For microglia and immune cells, the corresponding cell subsets were extracted for further subclustering analysis. Specifically, target cell populations were isolated in the Seurat package, followed by the repeated application of the standard analytical workflow, including data normalization, variable gene screening, data scaling, PCA-based dimensionality reduction, and clustering analysis (resolution = 0.4). Finally, these subpopulations were annotated based on their specific marker genes to clarify their cellular identities.

### 2.11. Significantly Dysregulated Genes Analysis and Enrichment Analysis

Differential expression analysis was performed using the FindMarkers function in the Seurat R package (v4.4.0). By default, the FindMarkers function uses the non-parametric Wilcoxon rank sum test for differential expression analysis. Fold changes (FCs) of genes were calculated as median (D3 or D7 or D14)/median (Control). The criteria of log_2_|FC| > 0.5 and adjusted *p*-value < 0.05 were used to screen the significant-expression genes. Common differentially expressed genes (DEGs) across all cell types were defined as those differentially expressed in every cell type examined. Cell type-specific DEGs were defined as those differentially expressed in only one single-cell type.

Gene Ontology (GO), Kyoto Encyclopedia of Genes and Genomes (KEGG), and Gene Set Enrichment Analysis (GSEA) were performed using the clusterProfiler R package (v4.8.1). The Mus musculus genome annotation database (org.Mm.eg.db) was used for gene ID conversion to ensure species-specific accuracy. Terms with a corrected *p*-value < 0.05 (FDR correction) were considered significantly enriched. Visualization was performed using the enrichplot package, including dotplots, barplots and gseaplot2, to illustrate key enriched pathways and their associated genes.

### 2.12. Pseudotime Analysis

Single-cell trajectory analysis and pseudotime inference were performed using Monocle2 (2.26.0), with the DDR-Tree algorithm employed for dimensionality reduction [24]. Before Monocle2 analysis, we selected cluster-specific marker genes identified from Seurat-based cell clustering and utilized raw count expression from high-quality cells that passed quality control filtering. Based on the constructed pseudotime trajectories, branch expression analysis modeling was subsequently applied to identify key genes that drive cell fate decisions at trajectory branch points.

### 2.13. CellChat

R package CellChat (version 2.1.0) was employed to investigate intercellular communication between cell clusters [25]. Specific intercellular interactions among distinct cell clusters were identified based on the ‘CellChatDB.mouse’ ligand–receptor database. Overexpressed genes and their corresponding interactions were identified using the identifyOverExpressedGenes and identifyOverExpressedInteractions functions. Cellular communication probabilities were calculated with the computeCommunProb function, and communication networks were aggregated using aggregateNet.

### 2.14. Statistical Analysis

GraphPad Prism 8 was used for data processing and analysis. All data are expressed as mean ± standard error of the mean (SEM). One-way analysis of variance was used, and *p*-values less than 0.05 were considered significant.

## 3. Results

### 3.1. Intracerebral Mycobacterial Infection Mouse Model

**Figure 1:**
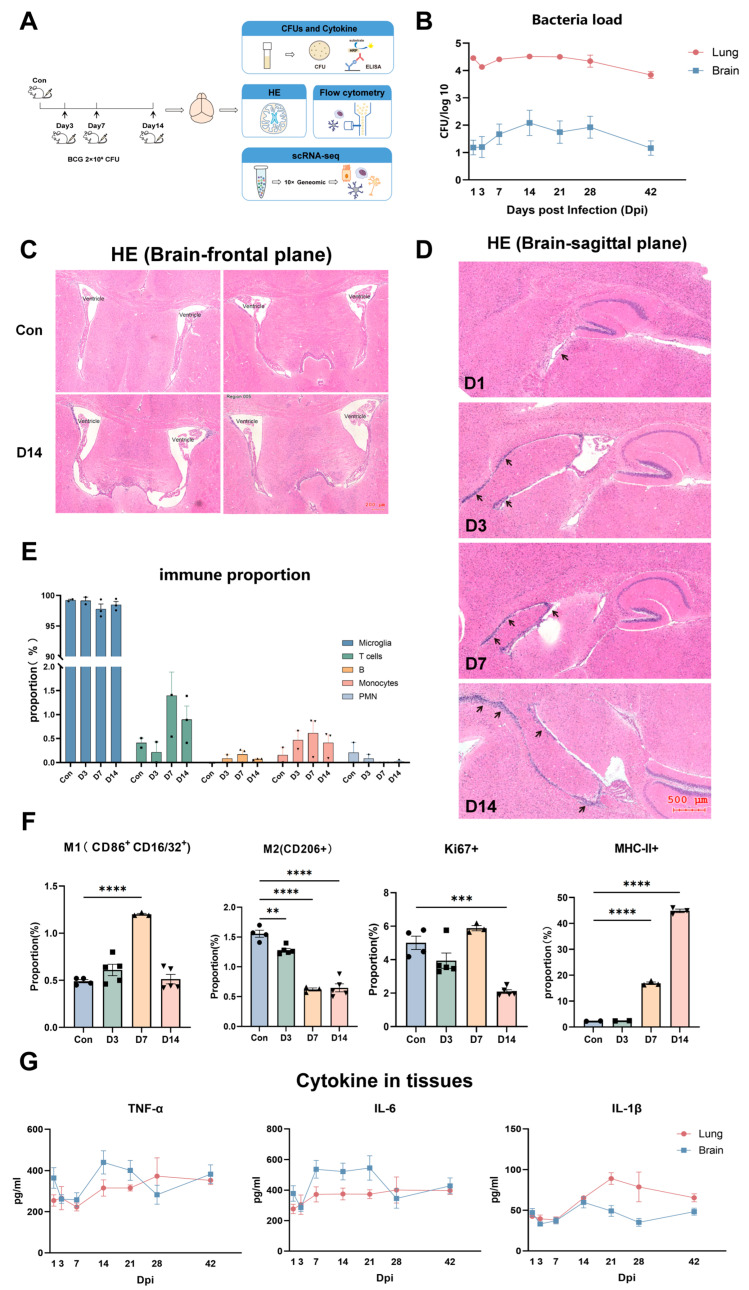
**Animal models of intracerebral mycobacterial infection.** (**A**) Schematic depiction of the experimental models that were used to study the pathogenesis and immune responses of intracerebral mycobacterial infection. (**B**) Line plot shows the CFUs in the lung and brain at different days post-infection. (**C**) Typical images of H&E staining of the frontal plane from the brains of infected mice at 14 dpi (*n* = 3) and control mice (*n* = 3) demonstrate ventricular enlargement. Scale bar: 200 μm. (**D**) Typical images of H&E staining of the sagittal plane from the brains of infected mice at 1, 3, 7, and 14 days post-BCG infection (D1, D3, D7, and D14) (*n* = 3 for each group) demonstrate influx and infiltration of immune cells (as indicated by the arrows). Scale bar: 500 μm. (**E**) Bar graph illustrates the change in the proportion of immune cells in brains in the control group (Con) and at 3, 7, and 14 days post-infection (D3, D7, and D14), including microglia, T cells, B cells, monocytes, and polymorphonuclear leukocytes. (**F**) Bar graphs illustrate the functional changes in microglia in the control group (Con) and at 3, 7, and 14 days post-infection (D3, D7, and D14). Data represent the results of two independent experiments, and graphs/dots represent the mean ± SEM of six mice. ** *p* < 0.01, *** *p* < 0.001, **** *p* < 0.0001. Statistical analysis was performed using one-way analysis of variance (ANOVA) followed by Bonferroni correction. (**G**) Line plots show dynamic changes in TNF-α, IL-6, and IL-1β in mouse lung (red) and brain (blue) tissues within 1 to 42 days post-infection. Graphs/dots represent the mean ± SEM of four mice.

To simulate hematogenous dissemination and to characterize cerebral inflammation, we established a mouse model via tail-vein intravenous BCG inoculation. Six-week-old BALB/c mice were injected with 2 × 10^6^ CFU BCG or an equal volume of PBS, and brains were harvested at successive time points (Figure 1A). We quantified the bacterial burden in tissues via agar-plate colony counting. The plates verified bacterial presence in the brain and lung tissues (Figure S1A), with cerebral bacterial load peaking at 3–14 dpi and gradually clearing thereafter (Figure 1B). Histopathology showed disorganized, detached choroid plexus and ependyma, enlarged ventricles (Figure 1C), and marked periventricular immune cell infiltration (Figure 1D); lungs displayed severe inflammation with alveolar architectural disruption (Figure S1B).

Flow cytometry revealed elevated infiltrating T cells and monocytes/macrophages in the brain, alongside reduced polymorphonuclear leukocytes (Figure 1E and Figure S1C). Microglia exhibited decreased proportion and proliferation, with M1 pro-inflammatory polarization and enhanced antigen presentation peaking at 14 dpi (Figure 1F and Figure S1D), consistent with cerebral bacterial load dynamics. Cerebral interleukin-1β (IL-1β), tumor necrosis factor-α (TNF-α), and interleukin-6 (IL-6) all peaked at 14 dpi and subsequently declined (Figure 1G).

Intravenous injection of BCG via the tail vein allowed for the successful establishment of a mouse model of intracerebral mycobacterial infection, characterized by both pulmonary infection and CNS involvement. Following hematogenous BCG infection, the cerebral immune microenvironment underwent dynamic remodeling, manifested by phenotypic switching of microglial function, infiltration of peripheral immune cells, and imbalance of the cytokine network; these changes constitute the immunopathological basis of intracerebral mycobacterial infection.

### 3.2. Dynamic and Temporal Reprogramming of the Intracerebral Immune Landscape During BCG Hematogenous Infection

**Figure 2:**
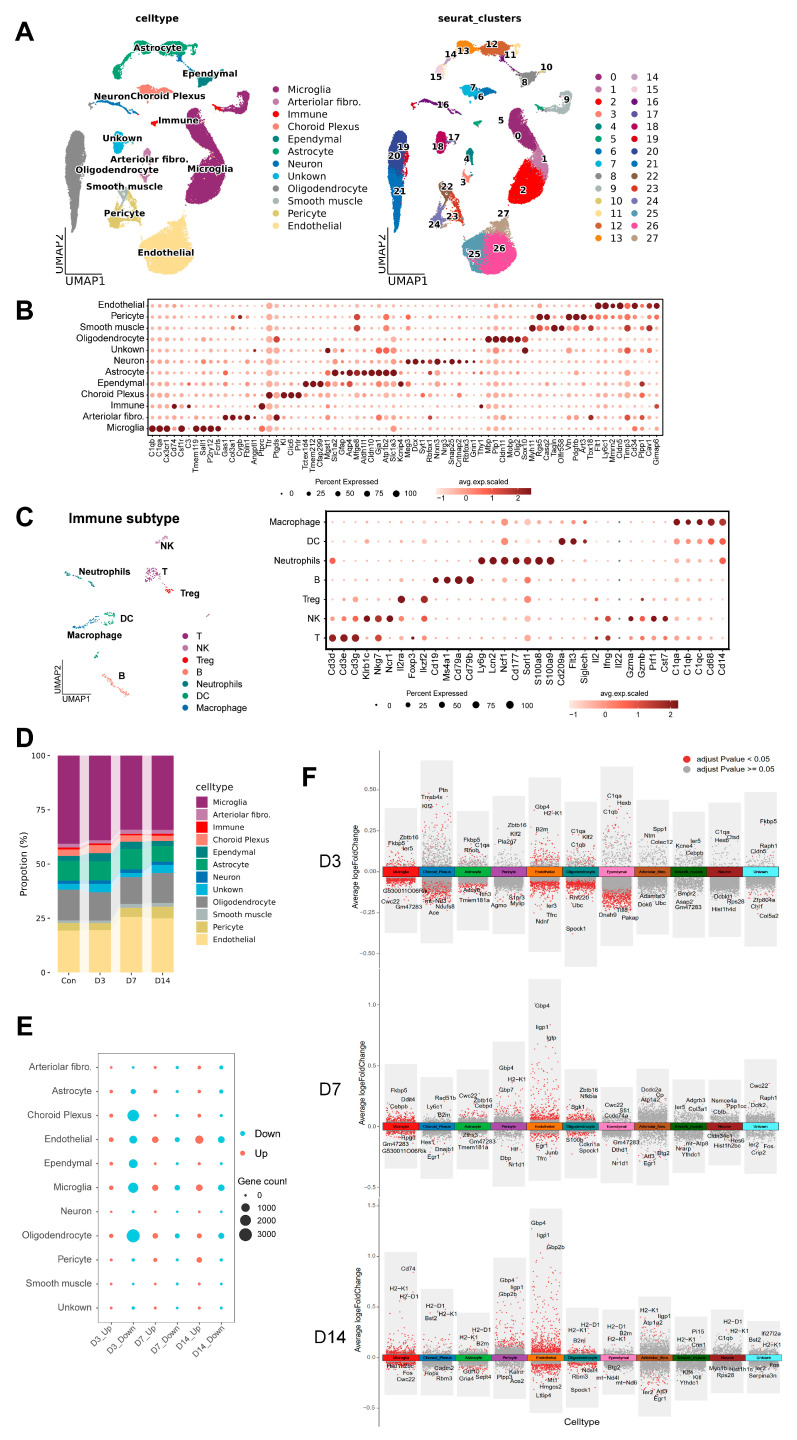
**Single-cell transcriptome atlas in intracerebral mycobacterial infection mouse model.** (**A**) Dimension reduction UMAP of 12 cell types. Different colors represent different cell types. (**B**) The expression intensity and specificity of markers in 12 major cell types. (**C**) Dimension reduction UMAP of re-clustering immune cells, including B cells, T cells, NK cells, Treg cells, macrophages and neutrophils (**Left**). The expression intensity and specificity of markers in immune cell types (**Right**). (**D**) The proportion distribution plot of 12 major cell types. (**E**) Bubble plot of the number of DEGs in each identified cell type at 3, 7, and 14 days post-infection (D3, D7, and D14) compared with the control group. Red dots represent upregulated genes (Up), blue dots represent downregulated genes (Down), and the size corresponds to the number of DEGs. (**F**) Volcano plots of DEGs in each cell subset at different time points post-infection relative to the control (Con). Red dots indicate significantly upregulated genes (adjusted *p*-value < 0.05), and gray dots indicate genes with no significant difference. Several top genes with the most significant expression changes in each subset are labeled. Differential expression testing is conducted using the non-parametric Wilcoxon rank-sum test.

To further characterize pathogenesis and immune responses, we performed 10× Genomics scRNA-seq on brains from PBS control (Con) and 3, 7, 14 dpi (D3/D7/D14) mice (Figure 1A). To reduce individual variation, six mouse brains were pooled per biological replicate, with two technical replicates per group (*n* = 2). After quality control (filtering low-expression cells and doublets), a total of 55,176 high-quality single-cells were retained. Based on highly expressed genes and published markers in the previous literature, we defined 12 major cell types in the brain and displayed their distribution using UMAP (Figure 2A,B). These encompassed brain-resident cells, structural cells of the vasculature and nervous tissue, and infiltrating immune cells derived from the periphery. The Unknown cluster consisted of heterogeneous precursor cells. Peripheral immune cells (Immune) were further resolved into distinct subsets based on their transcriptional profiles (Figure 2C). After infection, brain cellular composition shifted dynamically: microglia represented the most abundant cell type in the brain, and their proportion gradually declined. Concurrently, the relative proportions of immune cells, endothelial cells, and pericytes were correspondingly elevated (Figure 2D).

All cell types displayed varying transcriptomic changes (Figure 2E). Notably, endothelial cells, choroid plexus epithelial cells, ependymal cells, and microglia displayed the largest numbers of DEGs at 3 dpi, with a prominent predominance of downregulated genes, reflecting early suppression or dysregulation of functions. The number of upregulated genes in microglia and endothelial cells gradually increased over the course of infection, indicating sustained transcriptional reprogramming in these cells. Volcano plot analysis further confirmed that the differentially expressed genes in each cell subset showed clear time-dependent and cell-specific patterns (Figure 2F). At the early stage of infection, inflammation-related genes (including *C1qa*, *C1qb*, *H2-K1*, *B2m*, and *Ier5*) were unregulated in endothelial cells, choroid plexus epithelial cells, and ependymal cells. As infection progressed to D7 and D14, both the number and fold-change magnitude of DEGs increased markedly in microglia and endothelial cells, accompanied by significantly enhanced expression of pro-inflammatory factors and genes involved in antigen presentation. Strikingly, this immune activation was not limited to immune cells and barrier cells, but also detected in non-immune cells, including neurons (Figure 2E).

These results demonstrate that following BCG infection, diverse cell types within the brain participate in the central immune response through a sophisticated transcriptional regulatory network.

### 3.3. Activation of BBB- and BCSFB-Associated Cells in Early Infection and Initiation of Central Immune Response

Following systemic infection, the earliest and strongest intracerebral response arises from brain-barrier constituent cells, with endothelial cells, choroid plexus epithelial cells, and ependymal cells showing significant transcriptomic changes (Figure 2E).

**Figure 3:**
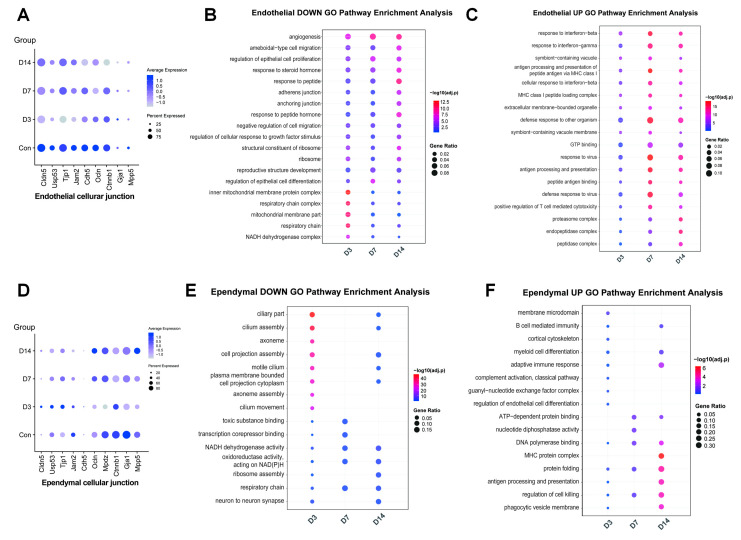
**Activation of endothelial cells and ependymal cells and initiation of central immune response.** (**A**) Dot plot showing the expression patterns of selected genes related to the cellular junction in endothelial cells. The size of each dot represents the proportion of cells expressing the gene, while the color intensity reflects the gene expression level. (**B**,**C**) Comparison of enriched functional terms for downregulated and upregulated DEGs in endothelial cells. (**D**) Dot plot showing the expression patterns of selected genes related to the cellular junction in ependymal cells. The size of each dot represents the proportion of cells expressing the gene, while the color intensity reflects the gene expression level. (**E**,**F**) Comparison of enriched functional terms for downregulated and upregulated DEGs in ependymal cells.

Traditionally, mycobacteria are considered to directly cross the BBB to invade the meninges and brain parenchyma, thereby triggering inflammatory responses [26]. The BBB is a collection of structural, transport, and metabolic barriers, which is formed by endothelial cells lining the blood vessels of the CNS through tight junctions and adherens junctions. A lot of intercellular tight junction proteins establish a high-resistance paracellular barrier, serving as a critical structure protecting the CNS from pathogen invasion [27]. Compared with the control group, endothelial cells underwent significant transcriptional reprogramming as early as 3dpi (D3). Among these changes, genes related to tight junctions, such as *Cldn5*, *Usp53*, *Tjp1*, *Jam2*, *Cdh5*, and *Ocln*, were downregulated (Figure 3A). In addition, genes associated with other cell junctions, including adherens junctions and anchoring junctions, were also downregulated, and their expression remained repressed at D7 and D14 (Figure 3A). GO functional enrichment analysis revealed that downregulated DEGs in endothelial cells were predominantly enriched in pathways involving vascular development, cell adhesion, and cell junction organization (Figure 3B), indicating that mycobacterial infection impaired endothelial cell junctions and barrier function of the BBB. However, it was not until D7 that endothelial cells began to substantially upregulate genes associated with leukocyte adhesion and extravasation, including chemokines (*Cxcl12*) (Figure S2A) and adhesion molecules (*Vcam1* and *Icam1*) (Figure S2B). Collectively, these changes suggest that endothelial cells exhibit a significant time-dependent pattern of immune activation. Meanwhile, their antigen processing and presenting, interferon response, and the response to pathogens peaked at D7 (Figure 3C), higher than those at D3 and D14, displaying a bell-shaped kinetic curve. This reflects a functional shift in endothelial cells from maintaining barrier homeostasis to actively participating in the central immune response following infection.

Additionally, some studies proposed that the BCSFB pathway is an important indirect invasion route [28]. After infection, ependymal cells exhibited unique transcriptomic changes characterized by the downregulation of genes related to cilia and cell junctions. At the initial stage (D3), it was observed that core genes involved in ciliary composition, assembly, and motility (such as *Foxj1* [29,30] and *Dnah* family genes [31,32]) were already downregulated in ependymal cells. Meanwhile, genes encoding intercellular junction protein genes (such as *Cldn11* and *Mdpz*) were also reduced (Figure 3D), suggesting that infection may disrupt the barrier integrity of the ependymal layer and its function in driving CSF flow. Furthermore, ependymal cells displayed enhanced adaptive immunity and antigen presentation (Figure 3E), indicating that ependymal cells can sense inflammatory signals within the CSF produced by the choroid plexus and infiltrating immune cells. Subsequently, ependymal cells mainly exhibited a stress state (Figure 3F). Moreover, they displayed a prominent antigen-presenting state during the peak of cerebral bacterial load, with significant upregulation of antigen-presenting genes (Figure 3F). As barrier and sensing cells, ependymal cells may serve as a critical gateway for transmitting inflammatory signals and bacteria from the CSF to the adjacent brain parenchyma (such as periventricular regions including the hippocampus and striatum), thereby influencing intracerebral immune responses.

### 3.4. Microglia Exhibit High Heterogeneity and Transdifferentiate into Disease-Associated States

**Figure 4:**
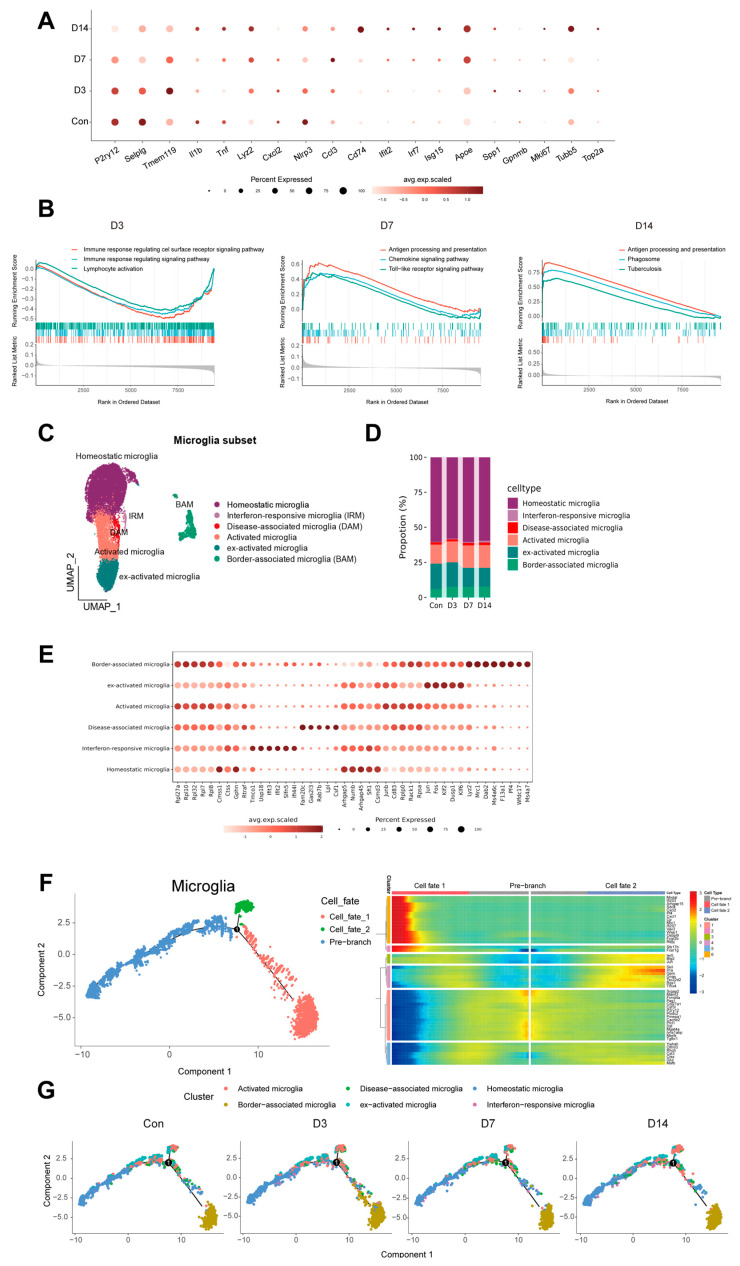
**Heterogeneity of microglia subsets.** (**A**) Dot plot showing the expression dynamics of selected functional genes of microglia from the control group (Con) and at 3, 7, and 14 days post-infection (D3, D7, and D14). The size of each dot represents the proportion of cells expressing the gene, and the color intensity reflects the gene expression level. (**B**) GSEA of microglia at D3, D7, and D14. The curve depicts the enrichment score, and the heatmap below shows the expression distribution of the core enriched genes. (**C**) Dimension reduction UMAP of re-clustering microglia. (**D**) Proportional distribution of microglial subsets. (**E**) The expression intensity and specificity of function markers in microglia subsets. (**F**) Single-cell trajectories of microglia by Monocle analysis (**left**). Heatmap overview of the most predictive genes along the inferred trajectory and selected feature plots (**right**). (**G**) Mapping of microglial subsets onto the pseudotime-ordered landscape.

Microglia, the brain-resident macrophages, are associated with brain development and homeostasis, immune recognition, defense, and clearance. As the primary targets of mycobacterial infection, microglia can recognize and phagocytose mycobacteria while participating in cytokine secretion and peripheral immune cell recruitment [33]. Gene expression analysis revealed that the expression of microglial homeostasis-related genes was gradually downregulated after infection, whereas the expression of genes associated with antigen presentation, inflammatory response, and interferon response was significantly upregulated. Notably, Apoe, a gene linked to neurodegenerative diseases, exhibited a marked upregulation (Figure 4A).

Gene Set Enrichment Analysis (GSEA) at distinct time points (Figure 4B) illuminated microglial functional dynamics: at D3, downregulated gene sets related to the regulation of immune response and lymphocyte activation, suggesting that they might be in the immune priming stage, actively suppressing immune surveillance and regulation to rapidly switch to the inflammatory response; at D7, antigen processing/presentation, chemokine, and TLR signaling were strongly upregulated, driving antigen-specific immunity and inflammatory amplification; by D14, microglia were significantly enriched in pathways related to antigen processing and presentation, as well as phagosome, further participating in antigen clearance. Collectively, these data define a staged transcriptional switch in microglia.

Moreover, microglia displayed high heterogeneity. We performed high-resolution re-clustering and trajectory inference on all microglia, focusing on their functional states, and identified six functional state clusters [17,34,35]. Based on core marker genes and differential pathways, these clusters were defined as follows: homeostatic microglia (high expression of *P2ry12* and *Tmem119*), interferon-responsive microglia (IRM, specifically high expression of *Isg15*, *Ifit3*, and *Ifit2*), disease-associated microglia (DAM, upregulation of *Apoe*, *Lpl*, and *Cst7*), activated microglia (high expression of *Cd74* and *MHC-II*, and core expression of *Il1b* and *Tnf*), border-associated microglia (BAM, high expression of *Mrc1* and *Ms4a7*), and exogenously activated microglia (high expression of *Fos*, *Jun*, and *Dusp1*), which are likely microglia abnormally activated during single-cell processing [36] (Figure 4C). After infection, the proportion of the homeostatic subset gradually decreased, while the proportions of DAM and IRM increased significantly (Figure 4D).

Pseudotime analysis revealed a dominant differentiation trajectory toward DAM (Figure 4F and Figure S3C,D): early infection triggered exit from homeostasis, branching into IRM, pro-inflammatory, and activated states, with convergence toward BAM; mid-to-late infection further consolidated into DAM, IRM, and BAM phenotypes (Figure 4G and Figure S2C). Microglial remodeling thus reflects staged functional shifts in the same population, not merely proportional subset changes.

### 3.5. Dynamic Intercellular Communication Networks Drive the Transition of Immune Responses

**Figure 5:**
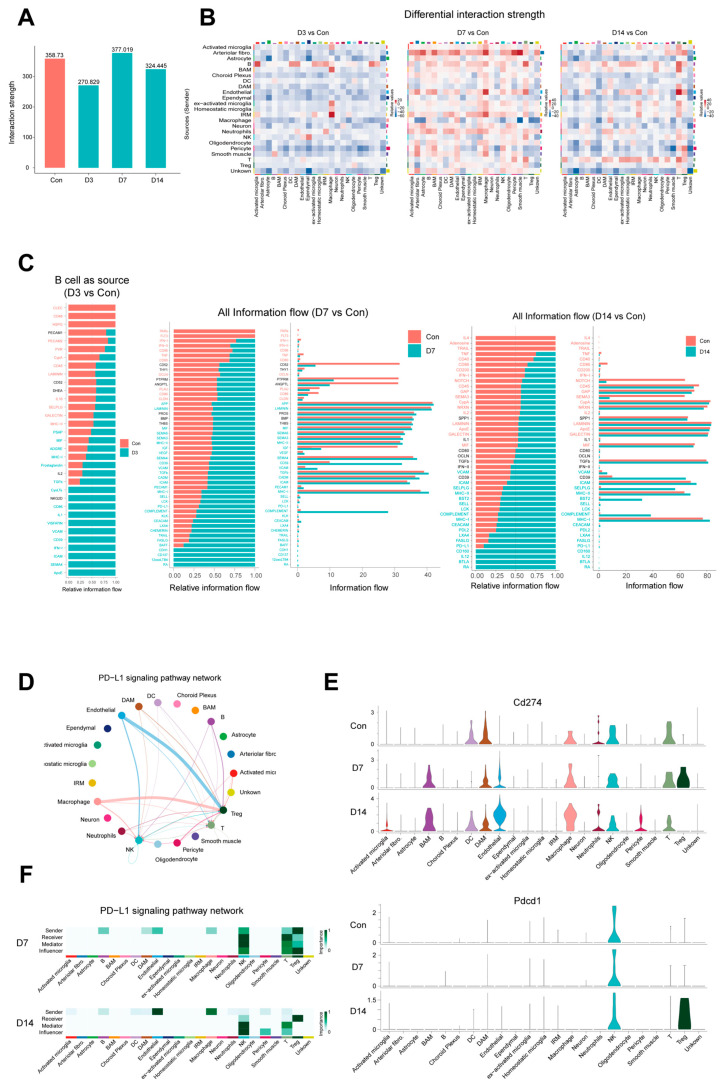
**Dynamic intercellular communication drives the transition of immune responses.** (**A**) Bar plot shows the change in communication strength. (**B**) Heatmaps display the change in communication intensity between different cell types compared to the control. (**C**) Bar plots show the differences in incoming information flow with B cells as the signaling source in D3 compared with the control group (**left**), and the differences in overall information flow within the cellular communication network of D7 (**middle**) and D14 (**right**) compared with the control group, with key signaling pathways ranked. Signaling pathways enriched in the control group are labeled in red, whereas those enriched in the infected group are labeled in green. (**D**) Intercellular communication network of the PD-L1 signaling pathway, showing the sending and receiving relationships of PD-L1 signals between various cell subsets after infection. The network is summarized by aggregating communication probabilities, and the thickness of the lines represents the communication strength. (**E**) Violin plots show the PD-L1 signaling genes expression pattern in the control group (Con) and at 7 and 14 dpi (D7 and D14). (**F**) Heatmap displays the network centrality scores of the PD-L1 signaling pathway at 7 and 14 dpi (D7, D14).

In addition to microglia, other immune cells (e.g., macrophages and T/B cells) participate in the immune response. To clarify how intercellular communication drives immune responses, we quantitatively analyzed cellular interaction networks at four time points using CellChat (Figure S3A). Results showed dynamic changes in total interaction intensity and network complexity post-infection: compared with the control, intensity slightly decreased at D3, peaked at D7, and declined at D14 (Figure 5A), reflecting the immune microenvironment progression from early activation and peak inflammation to late regulation and remodeling.

Compared with the control, global intercellular communication was attenuated at D3 (early stage), while immune cell function was enhanced (Figure 5B). Barrier cells (ependymal cells, choroid plexus epithelial cells, endothelial cells, and pericytes) showed the most significant reduction in outgoing communication, suggesting temporary homeostasis suppression or reprogramming. In contrast, B cells and partial microglia subsets served as early response hubs with increased outgoing communication (Figure S3B). Notably, B cells were activated early by the microenvironment, thereby secreting adhesion factors and cytokines for regulation and antigen presentation (Figure 5C-Left). Meanwhile, increased signal reception was concentrated in peripherally derived macrophages and IRM, indicating the rapid activation of innate immunity and the partial initiation of adaptive immunity.

At D7, outgoing/incoming communication of all major cell types was markedly enhanced, indicating that the immune microenvironment entered a highly active state. Signaling molecules, including semaphorin family molecules (SEMA3, SEMA4, and SEMA6), CHEMERIN, and VCAM/ICAM were upregulated; these molecules guided immune cell recruitment to infected regions. Additionally, MHC-I/II, COMPLEMENT, and KLK were directly involved in the initiation and amplification of inflammatory responses, while pathways like ICOS, CD137, BAFF, and NKG2D promoted antibody production and cellular immunity (Figure 5C-Middle and Figure S3C).

By D14, the immune microenvironment was characterized by enhanced outgoing and incoming signals of B/T cells (Figure 5B and Figure S3D). On the one hand, immune checkpoint signaling was globally upregulated, with the interaction strength of the PD-1-PD-L1 pathway reaching its peak. On the other hand, CDH1 and NCAM pathways mediated barrier function and tissue repair (Figure 5C-Right). These inhibitory signals, like PD-L1, collectively acted on activated T cells and NK cells, potentially suppressing their proliferation, effector functions, and cytokine production, thereby driving the transition of the immune microenvironment from aggressive inflammation to a state of persistent suppression and repair (Figure 5D). Ligand–receptor analysis showed Cd274 (PD-L1) was expressed in multiple immune cell subsets, and its expression was upregulated in myeloid subsets (especially activated microglia/macrophages). Notably, PD-L1 was not only highly expressed on immune cells but also showed an upregulation trend in endothelial cells (Figure 5E). In addition, in-depth analysis of regulatory T cells (Treg) demonstrated dynamic changes in immune checkpoint molecules during infection [37]: Tregs expressed Cd274 at D7, but shifted to high Pdcd1 (PD-1) expression at D14, while the expression of Cd274 returned to baseline (Figure 5E). Meanwhile, Treg transitioned from senders to receivers of the PD-1/PD-L1 signaling pathway (Figure 5F), indicating a shift in Treg-mediated immune suppression mechanisms by D14 (Figure S3E), which suggests a transition in the state of the immune response.

## 4. Discussion

In contrast to previous intracerebral inoculation models [33,38,39,40], we used tail-vein injection to simulate physiological hematogenous dissemination to the CNS. Intracardiac perfusion was employed to exclude blood bacterial contamination, and BCG was stably detected in the brain across all time points (1–42 dpi). Research on disseminated BCG disease remains extremely limited. The current understanding of the immune response triggered by intracerebral mycobacterial infection has been derived mainly from studies on TBM models. Both Mycobacterium tuberculosis and BCG belong to the genus Mycobacterium; they share commonalities in biological characteristics, host–pathogen interactions, and disease pathogenesis. Moreover, clinical and histologic manifestations of BCG infection (granulomatous inflammation and caseous necrosis) are similar to those of TB [41,42], and the brain pathological changes and partial clinical manifestations induced by intracranial dissemination of BCG are consistent with those of TBM [8]. Due to the absence of critical virulence factors, our study shows that BCG presented lower invasiveness and pathogenicity, with analogous cerebral bacterial load and inflammatory factor trends, but cerebral CFUs were one log lower than those of Mtb [43]. No cerebral granulomas or lesions formed in either group, while severe inflammatory infiltration was observed in the lungs.

Following infection, immune response was broadly activated across all cell types in the brain, extending beyond immune cells, characterized by elevated expression of genes involved in antigen processing and presentation, phagosomes, chemokines, and the complement system. During the early stage of infection, endothelial cells forming the BBB, and blood–CSF–brain barrier-associated choroid plexus epithelial cells/ependymal cells, all showed distinct profound responses. As a critical route in the pathogenesis of disseminated BCG disease, hematogenous dissemination leads to endothelial barrier dysfunction, inflammatory factor release, and peripheral immune cell recruitment, which in turn facilitates further bacterial invasion, immune cell infiltration, and subsequent parenchymal damage. Nonetheless, it remains unclear whether mycobacteria primarily reside within the brain parenchyma, vascular walls, or microvascular endothelial cells once it invades the CNS. However, combined with the vasogenic edema [8], in vitro endothelial invasion of BCG [44,45], and our finding of an early-peaking bell-shaped endothelial immune response, these jointly support the notion that mycobacteria initially localize at least within microvascular endothelial cells.

The blood–CSF–brain pathway is an identified indirect infectious route for CNS diseases, particularly relevant in infectious diseases. Barnacle has proposed that the blood–CSF–brain pathway may be one route of the Mtb invasion [46], and BCSFB disruption was confirmed in Mtb-infected mice [47]. BCGosis also manifests as CSF-adjacent lesions, CSF abnormalities, and radiological features (meningeal enhancement, ependymal injury, and hydrocephalus) [8]. Choroid plexus epithelial cells/ependymal cells (key BCSFB components) form essential physical barriers, participate in substance exchange, and mediate anti-pathogen immunity [48,49], which are all required to maintain CNS/CSF homeostasis. During CNS infection, these cells undergo various pathological alterations, such as ciliary thickening or loss, reduced beating frequency, and disrupted ultrastructure. These changes may increase the permeability of the CSF–brain barrier, enabling inflammatory factors and pathogens to accumulate and penetrate brain tissue, thereby leading to clinical manifestations, including hydrocephalus [50]. A scRNA-seq study of a BCG-injection model linked ependymal transcriptomic dysregulation (e.g., Frmd4a) to hydrocephalus and Alzheimer’s disease [13,14]. Ependymal cells actively participate in immunopathology [51], while IFN-γ was shown to mediate ependymal/choroid plexus dysfunction and induce ventricular enlargement [52], collectively indicating that choroid plexus epithelial cells and ependymal cells contribute substantially to the pathogenesis of intracerebral mycobacterial infection. Our data demonstrate that choroid plexus epithelial cells/ependymal cells were significantly activated at an early stage, displaying comparable antigen-presenting responses to endothelial cells, as well as ciliary dysfunction (downregulated structural/assembly genes), and reduced expression of junctional proteins. These findings support that the blood–CSF–brain barrier is a potential early invasion route for BCG entry into the CNS. Moreover, given that ependymal dysfunction is directly linked to disrupted CSF homeostasis and our observation of stage-specific ependymal states, CSF properties can be used as reliable indicators to reflect the state of pathology. Future host-directed therapies could integrate intrathecal administration to restore ependymal ciliary function, improving CSF circulation and mitigating hydrocephalus alongside anti-mycobacterial treatment.

Immune dysregulation—particularly the pro-inflammatory/anti-inflammatory balance—is central to TBM progression [46,53]. Thus, our temporal single-cell analysis analyzed the immune response in intracerebral mycobacterial infection, and uncovered three sequential CNS immune phases: barrier activation and innate immune recruitment (D3), robust inflammatory infiltration with integrated innate-adaptive immunity (D7), and pathogen clearance coupled with immune inhibition and tissue repair (D14) [33]. These phase-specific alterations are consistent with inflammatory factor dynamics. Microglia mediate the recognition, phagocytosis and clearance of mycobacteria, but their overactivation may trigger excessive inflammation and exacerbate brain lesions. Thus, their functional reprogramming drives immune phase transitions [54]. During the recruitment phase (D3), microglia suppress homeostatic surveillance to recruit macrophages and participate in IFN-mediated immune responses; then, they enhance antigen presentation and TLR signaling, further recruit peripheral immune cells, and coordinate innate and adaptive immunity to amplify and sustain the inflammatory storm in the inflammatory phase (D7). Finally, they prioritize phagocytic clearance in the regulatory/remodeling phase (D14). However, most patients with BCGosis suffer from immunodeficiencies, which impair one or multiple steps of the immune defense cascade. Such impairments include impaired bactericidal capacity of phagocytes, functional T-cell deficiency, and a dysregulated IFN-γ signaling pathway. These defects allow for unrestricted mycobacterial proliferation. Meanwhile, excessive or dysregulated inflammatory responses against BCG can also lead to severe tissue damage [7]. Defining these dynamic shifts in immune responses in intracerebral mycobacterial infection provides a mechanistic framework for how the CNS responds to mycobacterial invasion and the clinical pathogenesis of BCGosis, suggesting possible windows and targets for therapeutic intervention.

This study, however, has some limitations. First, our study mainly focuses on the immune changes from early infection, and discussions about chronic inflammation in the later stage of infection still need to be explored. Second, single-cell data lack spatial information, and cell–cell communication was computationally inferred, rather than experimentally validated. Third, the functional roles of identified cellular and molecular alterations require further confirmation by in vivo experiments and clinical samples.

In summary, during natural infection, mycobacteria may invade the CNS via both the BBB and the blood–CSF–brain route. Moreover, the CNS immune response to intracerebral mycobacterial infection undergoes a dynamic evolution characterized by a recruitment–inflammation–regulation/remodeling cascade, which lays a theoretical foundation for the development of stage-specific diagnostic biomarkers and temporally precise therapeutic strategies for intracerebral mycobacterial infection and disseminated BCG disease.

## Data Availability

The datasets generated during the current study are available from the corresponding authors on reasonable request.

## Supplementary Materials

The following supporting information can be downloaded at: https://www.mdpi.com/article/10.3390/pathogens15050531/s1, Figure S1: (A) The cultured M. bovis BCG colonies in brain at 3dpi, 7dpi, 14dpi. Each experiment was repeated three times. (B) Typical images of H&E staining from the lungs of infected mice at 14 dpi (*n* = 3) and control mice (*n* = 3) demonstrate severe inflammatory responses and disruption of the normal alveolar architectural structure. Scale bar: 100 μm. (C) Gating logic for immune cell phenotyping by flow cytometry. (D) Gating logic for microglial functional analysis by flow cytometry; Figure S2: (A) Dot plot showing the expression patterns of selected genes related to chemokines. The size of each dot represents the proportion of cells expressing the gene, while the color intensity reflects the gene expression level. (B) Dot plot showing the expression patterns of selected genes related to adhesion molecules. The size of each dot represents the proportion of cells expressing the gene, and the color intensity reflects the gene expression level. (C) Mapping and ordering of microglial subsets on pseudotime trajectories. (D) t-SNE plot of cell clustering distribution in principal components, color gradient represents pseudotime-based cell ordering; Figure S3: (A) Dot plot illustrating senders and receivers within inferred communication networks among all cell types across different infection time points. (B) Intercellular communication network changes (D3 vs. Con), summarized by aggregated communication probabilities (red: increased communication; blue: decreased communication). Left: B cells as senders interacting with other cells; Middle: Macrophages as receivers interacting with other cells; Right: Interferon responsive-related microglia as receivers interacting with other cells). (C) Bar plots show the differences in the information flow of immune cells and microglia (source and target, respectively) within the cellular communication network and rank important signaling pathways. The red top signaling pathways are enriched in the control group, while these greens are enriched in the D7 group. (D) Heatmap displays the change in communication intensity between different cells (D7 vs. D14). (E) Bar plots show the differences in the information flow of Treg (source and target, respectively) within the cellular communication network and rank important signaling pathways. The red top signaling pathways are enriched in the D14 group, while these greens are enriched in the D7 group.
