# Supplementary material for: Temporally Resolved Single-Cell RNA Sequencing Reveals Pathogenesis and Immune Responses in Intracerebral Bacille Calmette–Guérin (BCG) Infection
Source: Pathogens. 2026 May 14;15(5):531. doi: 10.3390/pathogens15050531 (PMC13209186; doi:10.3390/pathogens15050531)
Supplement: Supplementary file 1 [file pathogens-15-00531-s001.zip › Supplementary Material.pdf]

Supplementary Information

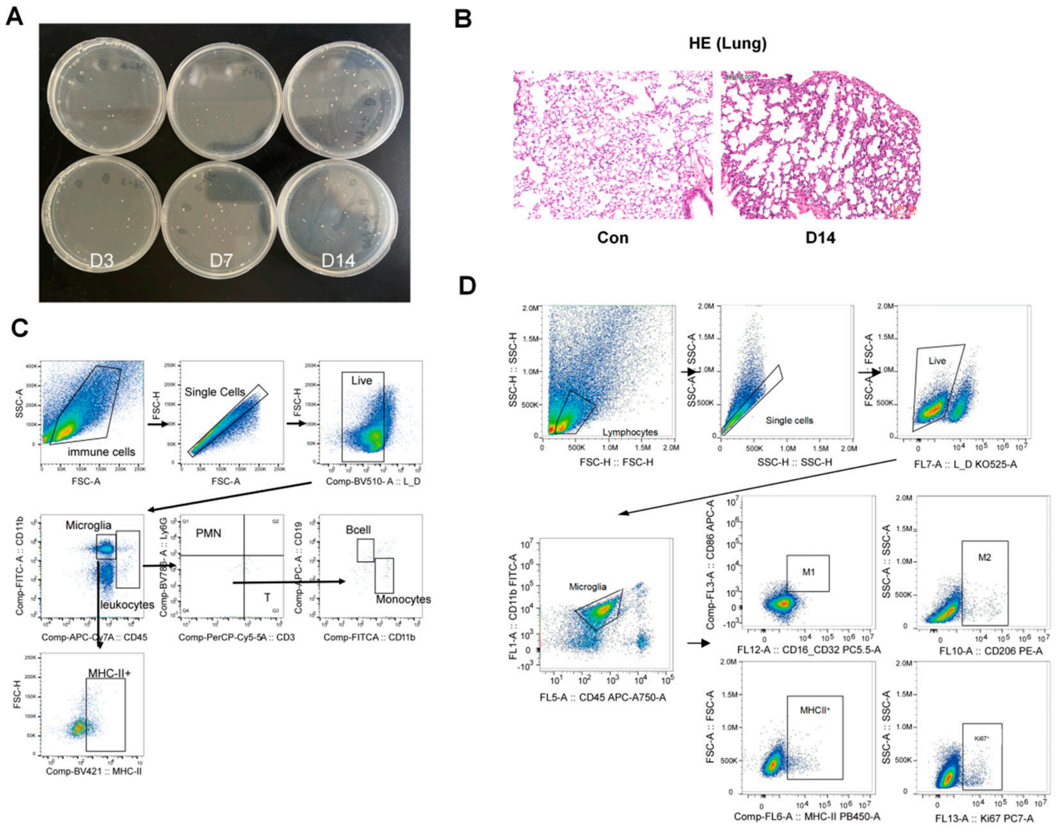

FigS1. (A) The cultured M. bovis BCG colonies in brain at 3dpi, 7dpi, 14dpi. Each experiment was repeated three times. (B) Typical images of H&E staining from the lungs of infected mice at 14 dpi (n = 3) and control mice (n = 3) demonstrate severe inflammatory responses and disruption of the normal alveolar architectural structure. Scale bar: 100 µm. (C) Gating logic for immune cell phenotyping by flow cytometry. (D) Gating logic for microglial functional analysis by flow cytometry.

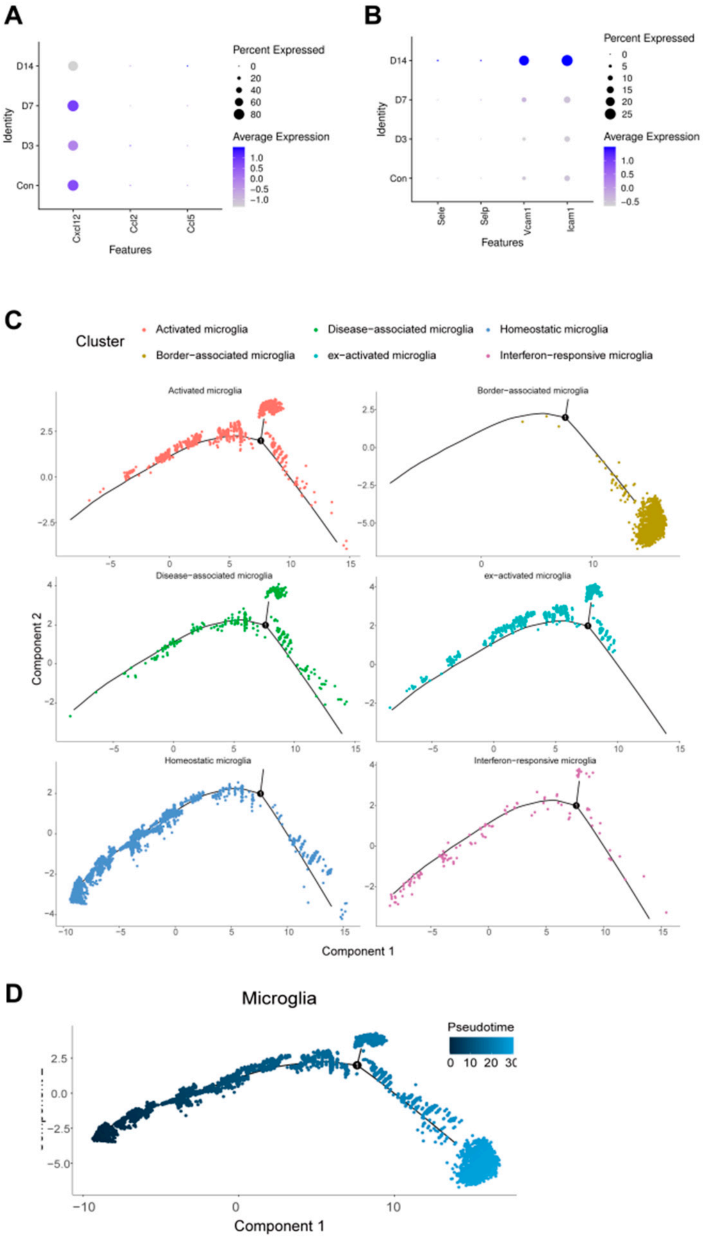

FigS2. (A) Dot plot showing the expression patterns of selected genes related to chemokines. The size of each dot represents the proportion of cells expressing the gene, while the color intensity reflects the gene expression level. (B) Dot plot showing the expression patterns of selected genes related to adhesion molecules. The size of each dot represents the proportion of cells expressing the gene, and the color intensity reflects the gene expression level. (C) Mapping and ordering of microglial subsets on pseudotime trajectories. (D) t-SNE plot of cell clustering distribution in principal components, color gradient represents pseudotime-based cell ordering

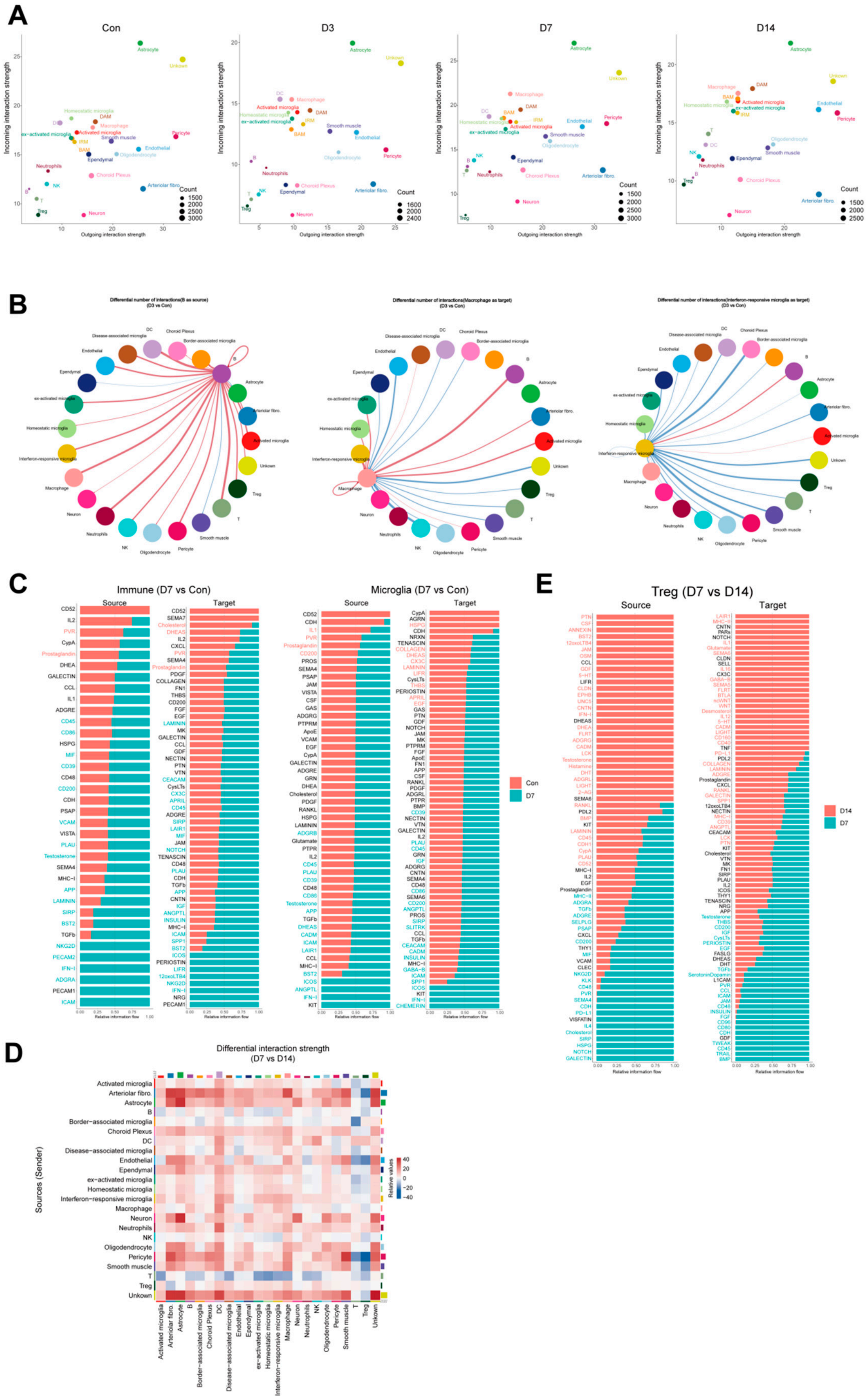
A
Con
D3
D7
D14
Incoming interaction strength
Outgoing interaction strength
Count
B
Differential number of interactions(B as source) (D3 vs Con)
Differential number of interactions(Macrophage as target) (D3 vs Con)
Differential number of interactions(Interferon-responsive microglia as target) (D3 vs Con)
C
Immune (D7 vs Con)
Microglia (D7 vs Con)
Source
Target
Relative information flow
Con
D7
D
Differential interaction strength (D7 vs D14)
Sources (Sender)
Relative values
E
Treg (D7 vs D14)
Source
Target
Relative information flow
D14
D7

FigS3. (A) Dot plot illustrating senders and receivers within inferred communication networks among all cell types across different infection time points. (B) Intercellular communication network changes (D3 vs Con), summarized by aggregated communication probabilities (red: increased communication; blue: decreased communication). Left: B cells as senders interacting with other cells; Middle: Macrophages as receivers interacting with other cells; Right: Interferon responsive-related microglia as receivers interacting with other cells). (C) Bar plots show the differences in the information flow of immune cells and microglia (source and target, respectively) within the cellular communication network and rank important signaling pathways. The red top signaling pathways are enriched in the control group, while these greens are enriched in the D7 group. (D) Heatmap displays the change in communication intensity between different cells (D7 vs D14). (E) Bar plots show the differences in the information flow of Treg (source and target, respectively) within the cellular communication network and rank important signaling pathways. The red top signaling pathways are enriched in the D14 group, while these greens are enriched in the D7 group.
