# Supplementary figures and images for: Temporally Resolved Single-Cell RNA Sequencing Reveals Pathogenesis and Immune Responses in Intracerebral Bacille Calmette–Guérin (BCG) Infection
Source: Pathogens. 2026 May 14;15(5):531. doi: 10.3390/pathogens15050531 (PMC13209186; doi:10.3390/pathogens15050531)

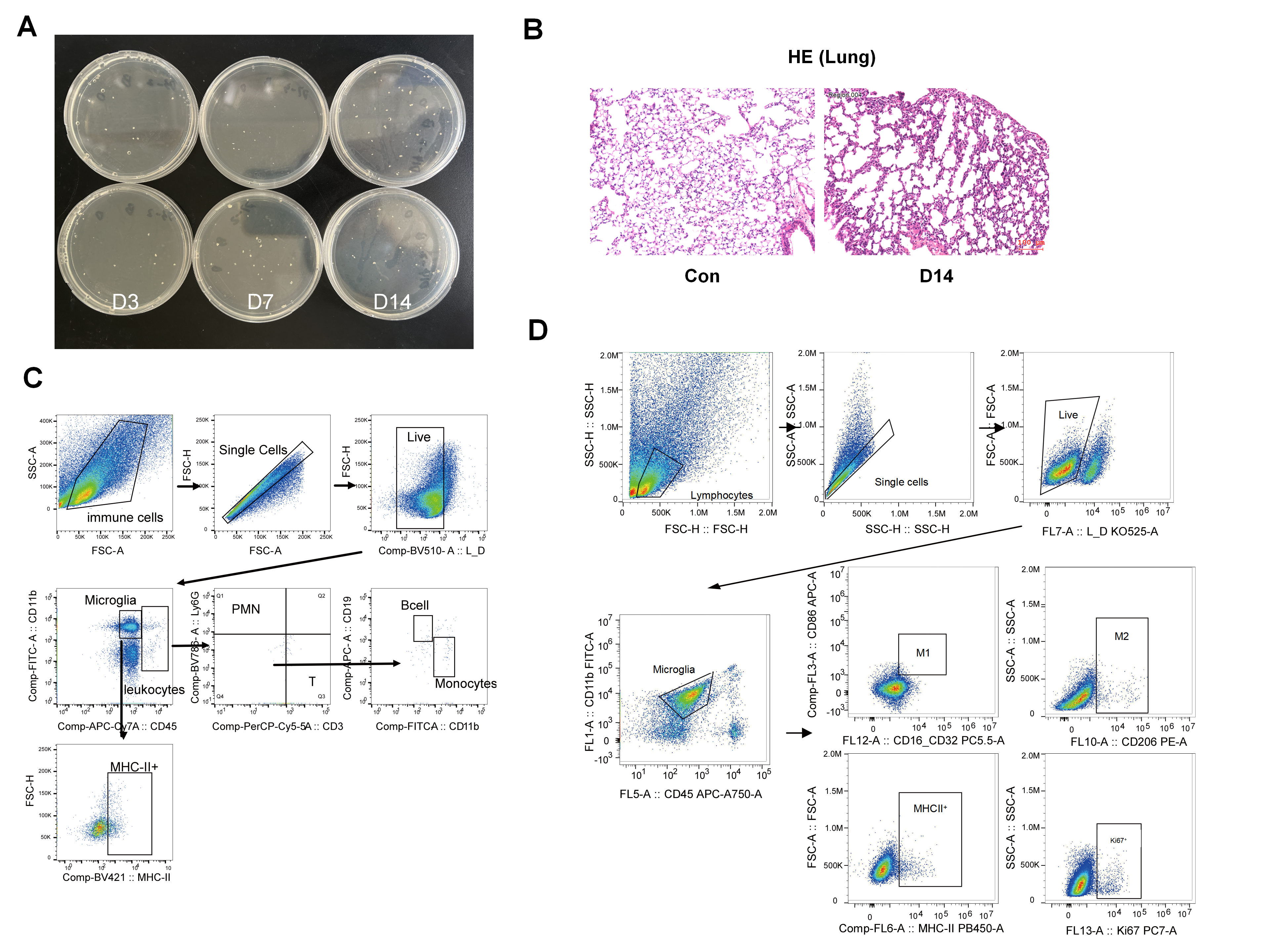

Supplement: Supplementary file 1 [file pathogens-15-00531-s001.zip › Supplement Figure_01.tif]
